# Healthy Diets to Prevent Obesity and Cardiovascular Diseases in Adolescents

**DOI:** 10.3390/nu16111740

**Published:** 2024-06-01

**Authors:** Anastasia Z. Kalea, Efstathia Papada

**Affiliations:** 1 Division of Medicine, University College London, London WC1E 6JF, UK; e.papada@ucl.ac.uk; 2 Institute of Cardiovascular Science, University College London, London WC1E 6HX, UK

Since the 1980s, there has been a global increase, decade by decade, in the rates of overweight and obesity among children, both in developed and developing countries [1]. This global issue has broad implications, including the development of psychiatric, psychological, and psychosocial problems during childhood as well as an elevated risk of suffering non-communicable diseases (NCDs) and of living with overweight and obesity in adulthood. Research shows that growth patterns during both the prenatal and postnatal stage are crucial in establishing the risk factors for obesity, while children from disadvantaged backgrounds experience higher rates of obesity, and this disparity has grown over the past decade [2].

Considering the urgency of this worldwide public health challenge, the member states of the World Health Organization (WHO) have embraced the objective of achieving “no increase in childhood overweight by 2025” as one of the six global nutrition goals outlined in the “Comprehensive Implementation Plan for Maternal, Infant, and Young Child Nutrition” [3]. This is in agreement with the target set for obesity and diabetes reduction between 2010 and 2025, as specified in the ‘WHO Global Action Plan for the Prevention and Control of Non-communicable Diseases 2013–2020’ [4,5]. The WHO estimates that, by 2025, a total—including adults and children—of approximately 167 million people will be living with a higher body weight. However, according to the 2022 World Obesity Atlas published by the World Obesity Federation, it is projected that, by 2030, approximately one billion individuals worldwide will be living with obesity [6]. The report highlights that not only will countries fail to achieve the 2025 WHO target of slowing the increase in obesity levels to the 2010 rates, but the global number of individuals with obesity will double across the globe.

Higher adiposity is often associated with significant short-term and long-term health consequences. In the short term, children who live with overweight or obesity often experience psychological stress linked to a variety of emotional, behavioural, and social difficulties, as well as more serious conditions, such as depression, anxiety, and low self-esteem [7]. It is also reported that, similarly to adults, children may face stigmatising behaviours and language from peers and educators in school as well as from parents and family members at home. To address this, several associations involved in obesity research recently issued a join statement championing the use of person-first language in childhood overweight and obesity [8]. As well as impacting mental health, it is well established that childhood and adolescent overweight and obesity have negative effects on physical health. Often, low-grade metabolic inflammation, which is characteristic of higher adiposity, is associated with comorbidities, such as asthma and liver complications and musculoskeletal problems [9], while children with overweight or obesity exhibit an increased presence of metabolic and cardiovascular risk factors. These can lead to early atherosclerosis and premature cardiovascular diseases (CVDs), including arterial hypertension and dyslipidaemia, as well as type 2 diabetes. However, unlike in the adult population, cardiovascular risk is not routinely assessed in the paediatric population, and there are limited data from studies on the key parameters linked to arterial stiffness in relation to eating behaviours and adiposity in this population group [10]. On the other hand, childhood obesity often persists into adulthood, driven by both physiological and behavioural factors, and the development of cardiometabolic diseases and their complications in adults is associated with disability and premature death.

Even though preventing obesity in the earliest stages of life offers a unique opportunity to disrupt the trajectory towards an unhealthy adulthood, addressing obesity in children is a complex challenge due to the limited effectiveness of the presently available treatments [11]. Successful efforts to address childhood obesity have included multifaceted family-centred programmes aimed at modifying behaviour. Often, such programmes have shown moderate short-term effects on weight-related outcomes, but they have generally proven challenging to implement, and their impact on cardiovascular risk factors is not as clearly established [10]. While supervised and structured exercise interventions have demonstrated improvements in various aspects, such as blood pressure, inflammation, carotid artery health, blood sugar regulation, cholesterol levels, and endothelial function, among children with obesity in the short term, the challenge lies in determining how to translate these interventions into sustainable long-term recommendations [12].

Promoting a healthy weight and optimal cardiometabolic health in children and adolescents is a multifaceted challenge that requires a combination of approaches and interventions. Research focusing on these areas is crucial to effectively treating childhood obesity and mitigating the associated cardiovascular disease risk factors, and thereby reducing the risk of cardiovascular disease development in adulthood. Our Special Issue invites original research papers and reviews that consider cardiometabolic health in their investigation into the effects of overweight and obesity in childhood, and approaches and interventions towards the prevention or treatment of these conditions. These may include nutrition education and counselling, involving schools or parents; family-based approaches; interventions in the school food environment; the promotion of physical activity programmes; behavioural interventions, such as cognitive behavioural therapy or mindful eating; or approaches that involve community activities, policy changes, or the involvement of healthcare services.

Successful interventions often involve a combination of these strategies and may need to be adapted to the specific needs and cultural contexts of the target population. A long-term commitment from governments, schools, healthcare providers, and communities is essential for creating a supportive environment for children and adolescents to achieve and maintain a healthy weight and optimal cardiometabolic health.
